# Neil and Smith’s Compendium of Medicine

**Published:** 1852-05

**Authors:** 


### NEIL AND SMITH'S COMPENDIUM OF MEDICINE.

We often hear the remark, "this is a wonderful age." It is an age of books, and especially of books for the young. How astonishingly are the juvenile publications multiplied; and not more increased in number than improved in quality. And, in our profession, how changed are things since the days of the pupilage of our fathers. The ponderous tomes have disappeared. There is now no committing Cheselden, Innis, or Cullen to memory. No long years of poring over the drowsy pages of Van Swieten and Boerhaave. Hippocrates, Galen, and Dioscorides stand neglected on the shelves of public libraries. Even Haller, Whytt, Cullen, Brown, and Darwin have yielded to youthful rivals, and a "compendious" generation of books has taken the place of the laborious systems of a by-gone age. We travel along the highways of learning by steam; professional knowledge is communicated by telegraph. For ourselves, we applaud the change; we confess we have no superstitious veneration for the *tempores acti.* Our conviction is deep and strong, that medical students were never favored before as are they of the living present time. The authors of this compend have rendered them a great service. It will prove a boon to them on the eve of their examination.

